# Lessons From LEADER – All-round Leadership

**DOI:** 10.17925/EE.2016.12.02.76

**Published:** 2016-08-28

**Authors:** Sanjay Kalra

**Affiliations:** Department of Endocrinology, Bharti Hospital, Karnal, India

**Keywords:** Cardiovascular outcomes, diabetes, empagliflozin, LEADER, liraglutide, lixisenatide, prevention

## Abstract

The large multinational, randomised, double-blind LEADER (Liraglutide Effect and Action in Diabetes: Evaluation of Cardiovascular Outcome Results - A Long Term Evaluation) trial recently reported the cardiovascular (CV) benefits achieved with liraglutide therapy in type 2 diabetes mellitus (T2DM). This editorial analyses the primary and secondary CV outcomes (CVO) results of the LEADER trial, and discusses the impact these will have on clinical practice of diabetes in specific, and medicine in general. It delves into the evolution of clinical and biochemical outcomes used in diabetes, and discusses the role of liraglutide in shaping future outcomes. The editorial describes the potential role of liraglutide in primary, secondary and tertiary prevention of CV disease (CVD), and suggests exaptation of this molecule for use in cardiology, nephrology and neurology.

The LEADER (Liraglutide Effect and Action in Diabetes: Evaluation of Cardiovascular Outcome Results - A Long Term Evaluation) trial recently reported the cardiovascular (CV) benefits achieved with liraglutide therapy in type 2 diabetes mellitus (T2DM). In a multinational, randomised, doubleblind trial involving 9340 participants, with a median follow-up of 3.8 years, various primary and secondary CV outcomes (CVO) were assessed. This editorial analyses the results of the LEADER trial, and discusses the impact these will have on clinical practice of diabetes in specific, and medicine in general.

## Study design

While the primary outcome of this time-to-event analysis was first occurrence of death from CV causes, nonfatal (including silent) myocardial infarction (MI), or nonfatal stroke, the trial also listed pre-specified exploratory outcomes. This included an expanded composite CV outcome (the three endpoints listed in the primary composite end point, coronary revascularisation, or hospitalisation for unstable angina pectoris or heart failure), death from any cause, a composite renal and retinal micro vascular outcome, neoplasms, and pancreatitis. The composite micro vascular outcome included nephropathy and retinopathy. Nephropathy was defined as the new onset of macro albuminuria, or doubling of serum creatinine level and an estimated glomerular filtration rate (eGFR) of ≤45 ml/minute /1.73m^2^, the need for continuous renal replacement therapy, or death from renal disease. Retinopathy was defined as the need for retinal photo coagulation or treatment with intra-vitreous agents, vitreous haemorrhage, or the onset of diabetes-related blindness.^1^

## Primary outcome

The primary composite outcome (first occurrence of death from CV causes, nonfatal MI, or nonfatal stroke) occurred in significantly fewer participants in the liraglutide group (608/4668; 13.0%) than in the placebo arm (694/4672; 14.9%) (hazard ratio [HR] 0.87; 95% confidence interval [CI] 0.78–0.97; p=0.01 for superiority). The major determinant of this benefit was the lower incidence of death from CV causes in the liraglutide group (219/4668; 4.7%) than in the placebo arm (278/4672; 6.0%) (HR 0.78; 95% CI 0.66-0.93; p=0.007). All-cause mortality was also lower in the liraglutide group (381/4668; 8.2%) than in the placebo arm (447/4672; 9.6%) (HR 0.85; 95% CI 0.74-0.97; p=0.02) (*Table 1*).^1^

## Secondary macrovascular outcome

While the incidence of nonfatal MI and nonfatal stroke was numerically lower in the liraglutide arm of the LEADER trial, as compared to the placebo group, the difference did not reach statistical significance. Nonfatal MI was reported in 281 out of 4668 liraglutide users (6.0%) as compared to 317 out of 4672 persons on placebo (6.8%) (HR 0.88; 95%CI 0.75-1.03; p=0.11). Similarly, nonfatal stroke occurred in 159 liraglutide users (3.4%) and 177 placebo-users (3.8%) (HR 0.89; 95% 0.72–1.11; p=0.30).^1^

There was no difference in the liraglutide and placebo arms with respect to hospitalisation for heart failure (HR 0.87; 95% CI 0.73–1.05; p=0.14).

## Microvascular outcome

The LEADER trial reported a significant reduction in the incidence of nephropathy (1.9 vs 1.5 events/patient years; p=0.003). There was however, no difference in the incidence of retinopathy (0.5 vs 0.6% events/patient years; p=0.33). The improvement in renal outcomes was driven predominantly by a lower rate of new-onset persistent macro albuminuria.^1^

## Comparison with other trials

The results from the LEADER trial compare favourably with those reported for lixisenatide by the ELIXA (Evaluation of Lixisenatide in Acute Coronary Syndrome) trial. In this trial, which enrolled patients with diabetes who had recently experienced an acute coronary syndrome event, lixisenatide was found to be safe, but not beneficial, for CVO.^2^ This difference may be attributed to the shorter half-life of lixisenatide, which may not be able to provide complete 24-hour glucagon-like peptide-1 (GLP1) activation.^1^

The results also differ from those of the EMPA-REG OUTCOME study, which analysed the CV effects of empagliflozin.^3^ The CV benefits of empagliflozin were evident within weeks of initiating therapy, while those of liraglutide were noted after 12–18 months of therapy. This suggests that empagliflozin has a haemodynamic or metabolic (pro-ketotic) mechanism of action,^4^ while liraglutide acts by modifying the progression of atherosclerotic disease. Further, liraglutide demonstrates improvement on all components of major adverse CV events (MACE), while empagliflozin was associated with an increase in the incidence of nonfatal stroke. This anomaly can be explained by the increase in haematocrit that is seen with sodium-glucose co-transporter-2 (SGLT2) inhibition.^5^

In the same manner as empagliflozin,^6^ liraglutide is an effective tertiary preventive strategy (preventing death due to CV events) rather than a primary preventive method (which will reduce chance of MI, stroke or heart failure).

## Safety and tolerability

Liraglutide proved to be safe and well tolerated during this long term study. Neoplasms and pancreatitis, which were pre-specified exploratory outcomes, did not increase with liraglutide use. This allays earlier (unfounded) fear mongering.^7^ Weight loss, lowering of systolic and diastolic blood pressure, and compensatory tachycardia occurred in the liraglutide group. Severe and confirmed hypoglycaemia were less common in the liraglutide group, in spite of better glucose control. This may be explained by the insulin sparing effect of liraglutide, which allowed lesser doses of insulin and insulin secretagogues to be used. All the differences noted above were statistically significant.

There is an added risk of gallstone disease, but it must be noted that cholelithiasis is precipitated by non-pharmacological weight lowering therapies as well.^8^

## Optimistic thoughts – expanded usage

The LEADER trial results encourage optimism, not only in diabetes care, but in other fields of medicine as well. The data put the spotlight on changing definitions of clinical outcomes. A century ago, prolonging the lives of children with T1DM by a few months, using starvation therapy, was the only treatment available: the measurable outcome, therefore, was the number of weeks or months of moribund existence achieved. With improvements in management, we moved to biochemical outcomes such as glycaemic control, and then added patient-centred outcomes such as quality of life to our targets. In the meantime, a shift from glucocentric intervention strategies to comprehensive metabolic care became evident. This evolution has now moved further ahead, including CVO as a desired goal in diabetes care. This has become possible with newer drugs such as liraglutide, which have demonstrated favourable effects on various clinical and vascular parameters.

**Table 1:** LEADER take home messages

Parameter	Key Message
MACE	Liraglutide reduced the risk for three-point MACE by 13% All three components of MACE (first occurrence of death from CV causes, nonfatal [including silent] MI, or nonfatal stroke) contributed to the risk reduction
Individual components of MACE	22% reduction in CV death 12% reduction in non-fatal MI 11% reduction in non-fatal stroke – first CV outcome study in diabetes segment to demonstrate reduction in non-fatal stroke
Other CV parameters	13% reduction in hospitalisation for heart failure 15% reduction in all-cause mortality
Microvascular complication: nephropathy	22% reduction in nephropathy, which was mainly driven by lower rates of new onset persistent macro-albuminuria
To prevent one MACE event	66 patients would need to be treated for 3 years with liraglutide
To prevent one death from any cause	98 patients would need to be treated for 3 years with liraglutide
Mechanism of benefit	Modification in progression of atherosclerotic vascular disease.
Summary	In patients with T2DM at high risk of CV events on standard therapy, liraglutide- as compared to placebo-treated patients had lower rates of CV events and all-cause death.

CV = cardiovascular, MACE = major adverse cardiovascular events, MI = myocardial infarction, T2DM = type 2 diabetes mellitus

Yet another evolutionary trend promoted by LEADER is the merging of macrovascular and microvascular health. Equally robust benefits of liraglutide on CV, cerebrovascular and renal outcomes suggest that these are all components of the same syndrome. It also suggests that timely intervention can modify the progression of vascular disease in diabetes. The beneficial effects appear evident at primary prevention (preventing onset of macro albuminuria), secondary prevention (prevention of worsening of renal function), and tertiary prevention (prevention of death). Similar to disease-modifying agents used in autoimmune diseases such as rheumatoid arthritis,^9^ liraglutide may herald the start of use of disease modifying anti-diabetic drugs (DMADs).

Such a use may find place in cardiology, nephrology and neurology, too, where high risk persons may benefit from the atherosclerosis modifying, morbidity attenuating, and mortality lowering properties of liraglutide.

## Unanswered questions

The LEADER trial does leave many questions unanswered, though. Are there any direct benefits of liraglutide in the kidney, especially on glomerular haemodynamics, which contribute to beneficial outcomes? How do we explain the non-significant numerical increase in retinopathy events? Is this some form of a pharmacologically induced or accentuated, reno-retinal dissociation? Do the CV benefits of liraglutide extend beyond 5 years, and will they be evident in persons at lower risk of CVD?

Recently, liraglutide has been approved as a weight loss agent, and can be prescribed to euglycaemic persons as well.^10^ Endocrine pharmacology is now being exapted for non-endocrine use.^11^ Can the results of LEADER be extrapolated to allow the use of liraglutide as a CV-protective or reno-protective therapy for non-diabetes patients? If so, should the drug be used in non-diabetes with established CVD or chronic kidney disease (CKD), or even in those at risk of CVD or CKD? Through future trials, we hope to have answers to these questions, hopefully in the affirmative.

## Summary

Liraglutide, thus has been able to demonstrate 360-degree benefits, in terms of reduction in risk of CVD, renal disease, and death due to all causes. These benefits are achieved in a safe and well tolerated manner, with no increase in risk of pancreatitis or neoplasms. We should be able to label LEADER, and liraglutide, as providing all-round leadership, not only in diabetes care, but in preventive cardiology and nephrology.
